# Supplementary material for: Brefeldin A—A Major Pathogenic Factor of Peanut Pod Rot from Fusarium neocosmosporiellum
Source: Toxins (Basel). 2024 Dec 18;16(12):548. doi: 10.3390/toxins16120548 (PMC11679824; doi:10.3390/toxins16120548)
Supplement: Supplementary file 1 [file toxins-16-00548-s001.zip › toxins-3304429-supplementary.pdf]

Supplementary files for

Brefeldin A, a Major Pathogenic Factor of Peanut Pod Rot from Fusarium neocosmosporiellum

Supplementary Data Contents

Figure S1 HR-ESI-MS spectrometry of brefeldin A

Figure S2. DEPT 135°spectrum of brefeldin A (CD_3OD)

Figure S3. ^1H-^1H COSY spectrum of brefeldin A (CD_3OD)

Figure S4. HMBC spectrum of brefeldin A (CD_3OD)

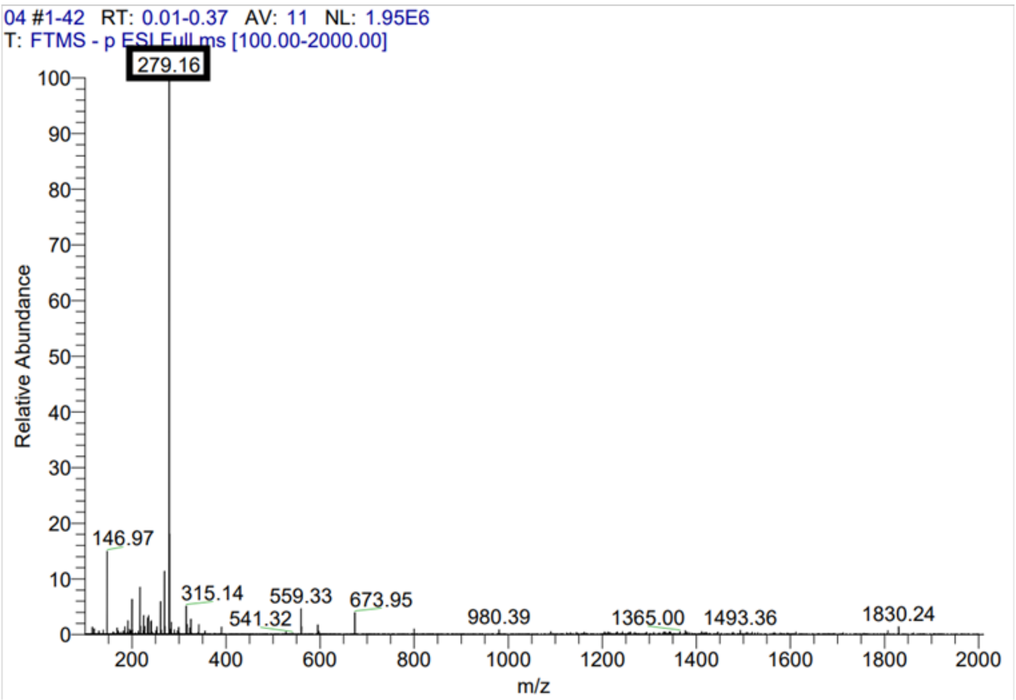

Figure S1. HR-ESI-MS spectrometry of brefeldin A

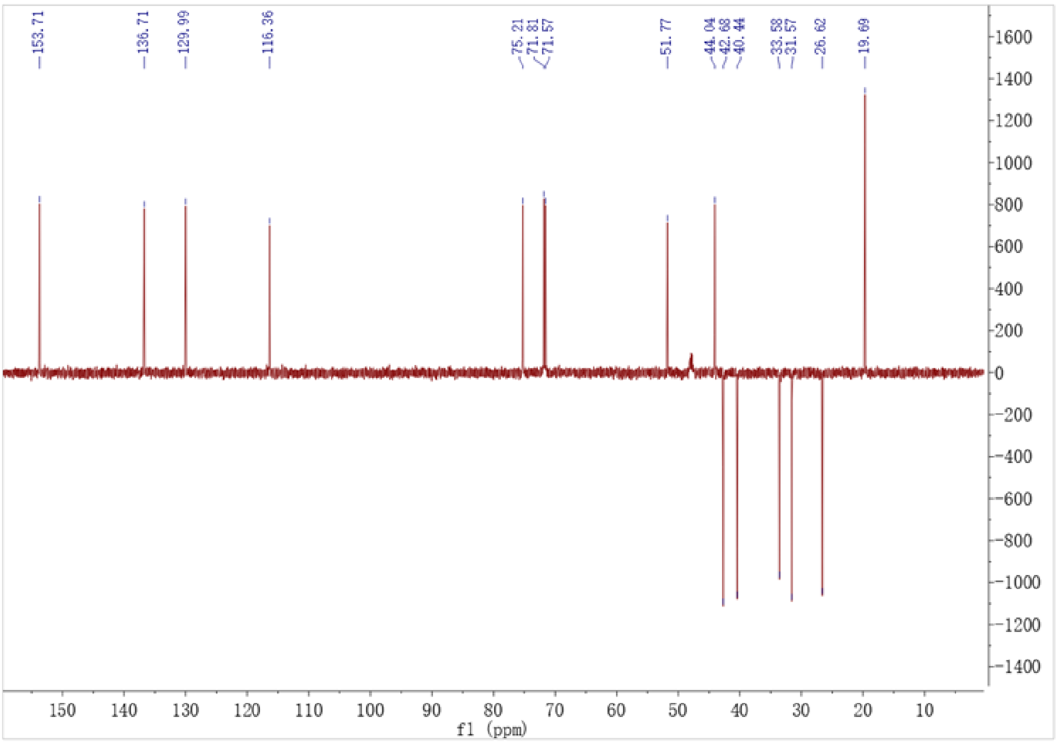

Figure S2. DEPT 135°spectrum of brefeldin A (CD_3OD)

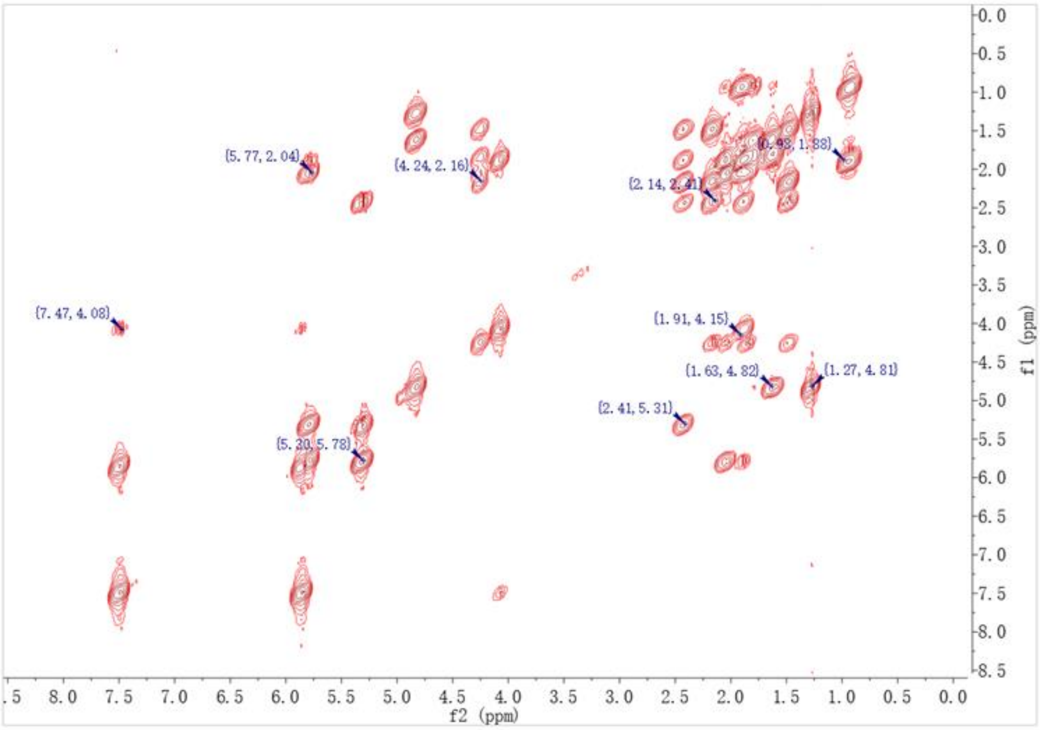

Figure S3. ^{1}H-^{1}H COSY spectrum of brefeldin A (CD_3OD)

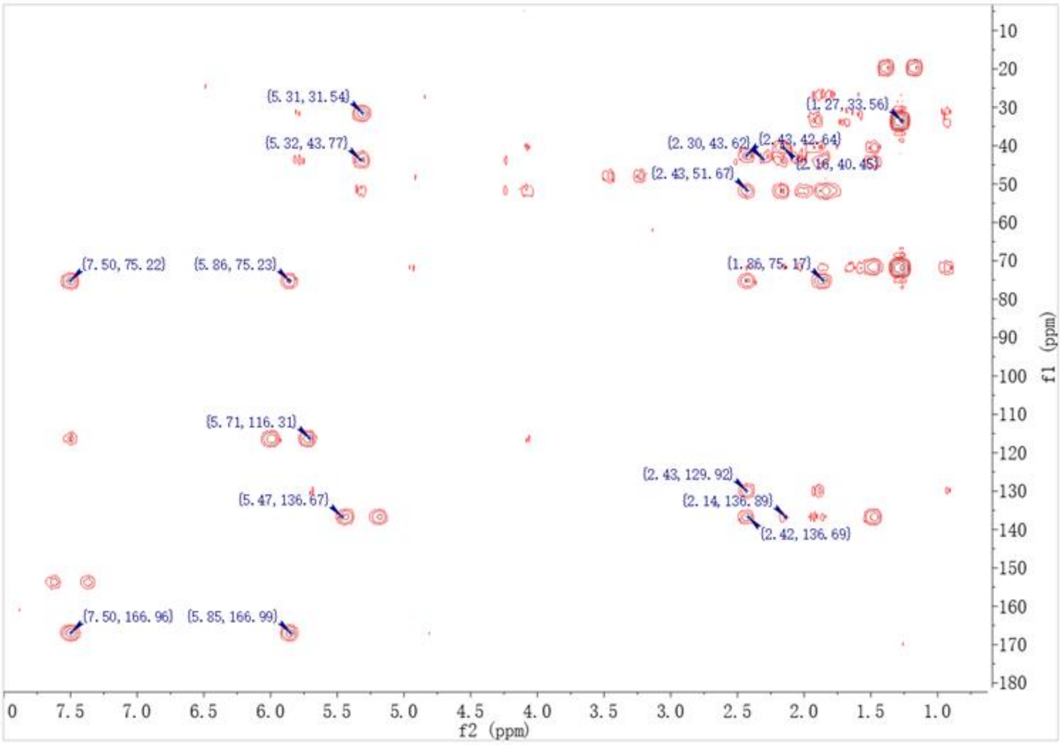

Figure S4. HMBC spectrum of brefeldin A (CD_3OD)
